# 4-[(5,5-Dimethyl-3-oxocyclo­hex-1-en­yl)amino]­benzene­sulfonamide

**DOI:** 10.1107/S1600536812029996

**Published:** 2012-07-07

**Authors:** Mansour S. Al-Said, Mostafa M. Ghorab, Hazem A. Ghabbour, Ching Kheng Quah, Hoong-Kun Fun

**Affiliations:** a Medicinal, Aromatic and Poisonous Plants Research Center (MAPPRC), College of Pharmacy, King Saud University, PO Box 2457, Riyadh 11451, Saudi Arabia; b Department of Pharmaceutical Chemistry, College of Pharmacy, King Saud University, Riyadh 11451, Saudi Arabia; c X-ray Crystallography Unit, School of Physics, Universiti Sains Malaysia, 11800 USM, Penang, Malaysia

## Abstract

In the title compound, C_14_H_18_N_2_O_3_S, the cyclo­hexene ring exhibits a distorted half-chair conformation and its mean plane makes a dihedral angle of 46.18 (8)° with the benzene ring. In the crystal, mol­ecules are linked *via* N—H⋯O, N—H⋯(O,O) and C—H⋯O hydrogen bonds, forming a three-dimensional network.

## Related literature

For general background to and the pharmacological activities of related compounds, see: Drews (2000 ▶); Supuran (2008 ▶); Supuran & Scozzafava (2000 ▶); Boyd (1988 ▶); Ghorab *et al.* (2007 ▶, 2009 ▶, 2011 ▶). For standard bond-length data, see: Allen *et al.* (1987 ▶). For ring conformations, see: Cremer & Pople (1975 ▶).
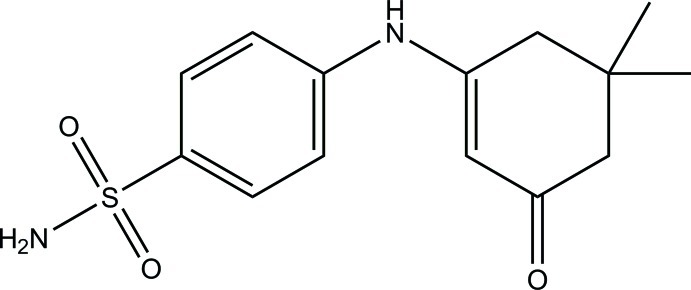


## Experimental

### 

#### Crystal data

C_14_H_18_N_2_O_3_S
*M* *_r_* = 294.36
Orthorhombic, 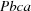

*a* = 11.0365 (3) Å
*b* = 13.4763 (3) Å
*c* = 20.0092 (6) Å
*V* = 2975.99 (14) Å^3^
*Z* = 8
Cu *K*α radiation
μ = 2.02 mm^−1^
*T* = 296 K
0.73 × 0.40 × 0.09 mm

#### Data collection

Bruker SMART APEXII CCD area-detector diffractometer
Absorption correction: multi-scan (*SADABS*; Bruker, 2009 ▶) *T*_min_ = 0.322, *T*_max_ = 0.839
10908 measured reflections
2829 independent reflections
2361 reflections with *I* > 2σ(*I*)
*R*_int_ = 0.036

#### Refinement

*R*[*F* ^2^ > 2σ(*F* ^2^)] = 0.044
*wR*(*F* ^2^) = 0.125
*S* = 1.04
2829 reflections
195 parameters
H atoms treated by a mixture of independent and constrained refinement
Δρ_max_ = 0.21 e Å^−3^
Δρ_min_ = −0.48 e Å^−3^

### 

Data collection: *APEX2* (Bruker, 2009 ▶); cell refinement: *SAINT* (Bruker, 2009 ▶); data reduction: *SAINT*; program(s) used to solve structure: *SHELXTL* (Sheldrick, 2008 ▶); program(s) used to refine structure: *SHELXTL*; molecular graphics: *SHELXTL*; software used to prepare material for publication: *SHELXTL* and *PLATON* (Spek, 2009 ▶).

## Supplementary Material

Crystal structure: contains datablock(s) global, I. DOI: 10.1107/S1600536812029996/is5161sup1.cif

Structure factors: contains datablock(s) I. DOI: 10.1107/S1600536812029996/is5161Isup2.hkl

Supplementary material file. DOI: 10.1107/S1600536812029996/is5161Isup3.cml

Additional supplementary materials:  crystallographic information; 3D view; checkCIF report

## Figures and Tables

**Table 1:** Hydrogen-bond geometry (Å, °)

*D*—H⋯*A*	*D*—H	H⋯*A*	*D*⋯*A*	*D*—H⋯*A*
N1—H1N1⋯O1^i^	0.88 (2)	2.10 (2)	2.958 (2)	166 (2)
N2—H1N2⋯O3^ii^	0.89 (2)	2.02 (2)	2.900 (2)	169 (2)
N2—H2N2⋯O3^iii^	0.88 (2)	2.13 (2)	2.969 (2)	159 (2)
C6—H6*A*⋯O2^iv^	0.97	2.44	3.229 (2)	139

Symmetry codes: (i) 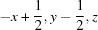; (ii) 
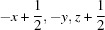
; (iii) 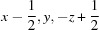; (iv) 
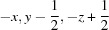
.

**Fig. 1.**
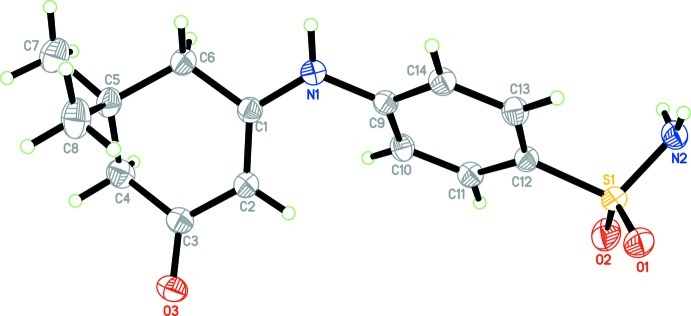
The molecular structure of the title compound showing 30% probability displacement ellipsoids for non-H atoms.

**Fig. 2.**
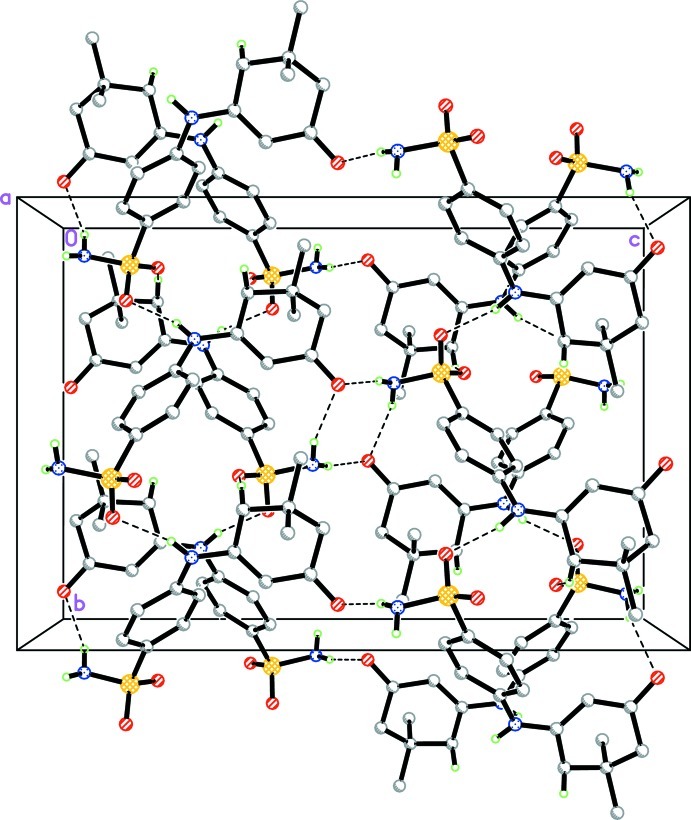
The crystal structure of the title compound, viewed along the a axis. H atoms not involved in hydrogen bonds (dashed lines) have been omitted for clarity.

## supplementary crystallographic information

### Comment

From literature survey, it was found that sulfonamides constitute an important class of drugs with several types of pharmacological activities including antibacterial (Drews, 2000), anti-carbonic anhydrase (Supuran, 2008), diuretic (Supuran & Scozzafava, 2000) and hypoglycemic (Boyd, 1988) properties. Also, some structurally novel sulfonamide derivatives have recently been reported to show substantial antitumor activity (Ghorab *et al.*, 2011). Based on the above informations and due to our interest in the synthesizing novel sulfonamides (Ghorab *et al.*, 2009), the present investigation deals with the design and synthesis of a novel 4-(5,5-dimethyl-3- oxocyclohex-1-enylamino) carrying a biologically active sulfonamide moiety for evaluation as an anticancer agent.

In the title molecule (Fig. 1), the cyclohexene ring (C1–C6) exhibits a distorted half-chair conformation with puckering parameters (Cremer & Pople, 1975) Q = 0.4561 (19) Å, Θ = 52.5 (2)° and φ = 210.9 (3) Å, and its least square plane makes a dihedral angle of 46.18 (8)° with the benzene ring (C9–C14). Bond lengths (Allen *et al.*, 1987) and angles are within normal ranges. In the crystal (Fig. 2), molecules are linked *via* intermolecular N1—H1N1···O1, N2—H1N2···O3, N2—H2N2···O3 and C6—H6A···O2 hydrogen bonds (Table 1), forming a three-dimensional network.

### Experimental

4-(5,5-Dimethyl-3-oxocyclohex-1-enylamino)benzenesulfonamide was prepared according to the previously reported procedure (Ghorab *et al.*, 2007). Single crystals suitable for an X-ray structural analysis was obtained by slow evaporation from ethanol at room temperature.

### Refinement

The N-bound hydrogen atoms was located in a difference Fourier map and refined freely [N—H = 0.88 (2)–0.89 (2) Å]. The remaining hydrogen atoms were positioned geometrically (C—H = 0.96–0.97 Å) and were refined using a riding model, with *U*_iso_(H) = 1.2 or 1.5*U*_eq_(C). A rotating group model was applied to the methyl groups.

### Crystal data

**Table:**

C_14_H_18_N_2_O_3_S	*F*(000) = 1248
*M**_r_* = 294.36	*D*_x_ = 1.314 Mg m^−^^3^
Orthorhombic, *P**b**c**a*	Cu *K*α radiation, λ = 1.54178 Å
Hall symbol: -P 2ac 2ab	Cell parameters from 2253 reflections
*a* = 11.0365 (3) Å	θ = 4.4–70.1°
*b* = 13.4763 (3) Å	µ = 2.02 mm^−^^1^
*c* = 20.0092 (6) Å	*T* = 296 K
*V* = 2975.99 (14) Å^3^	Plate, colourless
*Z* = 8	0.73 × 0.40 × 0.09 mm

### Data collection

**Table:**

Bruker SMART APEXII CCD area-detector diffractometer	2829 independent reflections
Radiation source: fine-focus sealed tube	2361 reflections with *I* > 2σ(*I*)
Graphite monochromator	*R*_int_ = 0.036
φ and ω scans	θ_max_ = 72.0°, θ_min_ = 4.4°
Absorption correction: multi-scan (*SADABS*; Bruker, 2009)	*h* = −11→13
*T*_min_ = 0.322, *T*_max_ = 0.839	*k* = −16→10
10908 measured reflections	*l* = −24→24

### Refinement

**Table:**

Refinement on *F*^2^	Primary atom site location: structure-invariant direct methods
Least-squares matrix: full	Secondary atom site location: difference Fourier map
*R*[*F*^2^ > 2σ(*F*^2^)] = 0.044	Hydrogen site location: inferred from neighbouring sites
*wR*(*F*^2^) = 0.125	H atoms treated by a mixture of independent and constrained refinement
*S* = 1.04	*w* = 1/[σ^2^(*F*_o_^2^) + (0.0919*P*)^2^] where *P* = (*F*_o_^2^ + 2*F*_c_^2^)/3
2829 reflections	(Δ/σ)_max_ = 0.001
195 parameters	Δρ_max_ = 0.21 e Å^−^^3^
0 restraints	Δρ_min_ = −0.48 e Å^−^^3^

### Special details

**Table:**

Geometry. All esds (except the esd in the dihedral angle between two l.s. planes) are estimated using the full covariance matrix. The cell esds are taken into account individually in the estimation of esds in distances, angles and torsion angles; correlations between esds in cell parameters are only used when they are defined by crystal symmetry. An approximate (isotropic) treatment of cell esds is used for estimating esds involving l.s. planes.
Refinement. Refinement of F^2^ against ALL reflections. The weighted R-factor wR and goodness of fit S are based on F^2^, conventional R-factors R are based on F, with F set to zero for negative F^2^. The threshold expression of F^2^ > 2sigma(F^2^) is used only for calculating R-factors(gt) etc. and is not relevant to the choice of reflections for refinement. R-factors based on F^2^ are statistically about twice as large as those based on F, and R- factors based on ALL data will be even larger.

### Fractional atomic coordinates and isotropic or equivalent isotropic displacement parameters (Å^2^)

**Table:**

	*x*	*y*	*z*	*U*_iso_*/*U*_eq_	
S1	−0.00703 (4)	0.12128 (3)	0.35905 (2)	0.04078 (17)	
N1	0.30747 (13)	−0.19577 (10)	0.24788 (7)	0.0409 (3)	
N2	−0.04392 (17)	0.09709 (12)	0.43487 (8)	0.0470 (4)	
O1	0.06697 (14)	0.20885 (9)	0.36157 (7)	0.0578 (4)	
O2	−0.11492 (13)	0.12253 (11)	0.31954 (8)	0.0628 (4)	
O3	0.33684 (13)	−0.09069 (10)	0.02398 (6)	0.0542 (4)	
C1	0.33333 (14)	−0.20944 (11)	0.18227 (8)	0.0371 (3)	
C2	0.31006 (16)	−0.14052 (12)	0.13444 (8)	0.0411 (4)	
H2A	0.2760	−0.0801	0.1467	0.049*	
C3	0.33664 (15)	−0.15894 (12)	0.06597 (9)	0.0419 (4)	
C4	0.36612 (17)	−0.26403 (13)	0.04640 (9)	0.0480 (4)	
H4A	0.4044	−0.2642	0.0028	0.058*	
H4B	0.2917	−0.3020	0.0432	0.058*	
C5	0.45074 (16)	−0.31347 (13)	0.09715 (9)	0.0440 (4)	
C6	0.39348 (16)	−0.30700 (12)	0.16681 (9)	0.0429 (4)	
H6A	0.3338	−0.3594	0.1711	0.052*	
H6B	0.4560	−0.3190	0.1999	0.052*	
C7	0.4679 (2)	−0.42291 (16)	0.07882 (12)	0.0677 (6)	
H7A	0.5099	−0.4277	0.0369	0.102*	
H7B	0.3902	−0.4544	0.0751	0.102*	
H7C	0.5145	−0.4553	0.1130	0.102*	
C8	0.57395 (17)	−0.26183 (16)	0.09661 (12)	0.0615 (5)	
H8A	0.6078	−0.2649	0.0525	0.092*	
H8B	0.6273	−0.2945	0.1275	0.092*	
H8C	0.5641	−0.1937	0.1096	0.092*	
C9	0.23173 (14)	−0.12110 (11)	0.27412 (8)	0.0356 (3)	
C10	0.12181 (15)	−0.09880 (12)	0.24388 (8)	0.0402 (4)	
H10A	0.0975	−0.1328	0.2057	0.048*	
C11	0.04870 (14)	−0.02559 (12)	0.27089 (8)	0.0392 (4)	
H11A	−0.0248	−0.0100	0.2507	0.047*	
C12	0.08487 (14)	0.02434 (11)	0.32787 (8)	0.0365 (3)	
C13	0.19250 (15)	0.00026 (12)	0.35958 (8)	0.0400 (4)	
H13A	0.2153	0.0332	0.3984	0.048*	
C14	0.26572 (15)	−0.07305 (12)	0.33301 (8)	0.0401 (4)	
H14A	0.3375	−0.0903	0.3544	0.048*	
H1N1	0.3431 (18)	−0.2337 (16)	0.2774 (11)	0.058 (6)*	
H1N2	0.024 (2)	0.1016 (15)	0.4588 (12)	0.053 (6)*	
H2N2	−0.090 (2)	0.0441 (18)	0.4374 (12)	0.070 (7)*	

### Atomic displacement parameters (Å^2^)

**Table:**

	*U*^11^	*U*^22^	*U*^33^	*U*^12^	*U*^13^	*U*^23^
S1	0.0482 (3)	0.0360 (3)	0.0381 (3)	0.00670 (16)	0.00260 (16)	0.00170 (14)
N1	0.0455 (7)	0.0400 (7)	0.0372 (7)	0.0075 (6)	0.0036 (6)	0.0035 (6)
N2	0.0552 (10)	0.0451 (8)	0.0408 (8)	−0.0019 (7)	0.0078 (7)	−0.0014 (6)
O1	0.0801 (10)	0.0362 (6)	0.0572 (8)	−0.0043 (6)	0.0143 (7)	0.0014 (5)
O2	0.0568 (8)	0.0722 (9)	0.0595 (9)	0.0265 (7)	−0.0102 (7)	−0.0065 (7)
O3	0.0713 (9)	0.0505 (7)	0.0409 (7)	0.0019 (6)	0.0039 (6)	0.0088 (5)
C1	0.0363 (8)	0.0355 (8)	0.0394 (8)	−0.0015 (6)	0.0039 (6)	−0.0001 (6)
C2	0.0468 (9)	0.0351 (8)	0.0414 (8)	0.0041 (7)	0.0056 (7)	0.0007 (6)
C3	0.0419 (8)	0.0429 (9)	0.0408 (8)	−0.0026 (7)	0.0013 (7)	0.0026 (7)
C4	0.0592 (11)	0.0442 (9)	0.0408 (8)	−0.0062 (8)	−0.0010 (8)	−0.0059 (7)
C5	0.0474 (9)	0.0390 (8)	0.0456 (9)	0.0014 (7)	0.0044 (7)	−0.0092 (7)
C6	0.0458 (9)	0.0373 (8)	0.0457 (9)	0.0044 (7)	0.0046 (7)	0.0002 (7)
C7	0.0897 (16)	0.0462 (11)	0.0673 (13)	0.0112 (11)	0.0052 (12)	−0.0157 (10)
C8	0.0440 (10)	0.0679 (12)	0.0724 (13)	−0.0023 (9)	0.0105 (9)	−0.0133 (10)
C9	0.0369 (8)	0.0343 (8)	0.0357 (8)	−0.0009 (6)	0.0055 (6)	0.0031 (5)
C10	0.0407 (8)	0.0431 (8)	0.0368 (8)	−0.0027 (7)	−0.0003 (6)	−0.0054 (6)
C11	0.0358 (8)	0.0425 (8)	0.0392 (8)	0.0002 (7)	−0.0021 (6)	0.0007 (6)
C12	0.0382 (8)	0.0346 (7)	0.0366 (8)	−0.0002 (6)	0.0042 (6)	0.0002 (6)
C13	0.0398 (8)	0.0424 (8)	0.0379 (8)	−0.0020 (7)	−0.0023 (6)	−0.0043 (6)
C14	0.0358 (8)	0.0458 (9)	0.0386 (8)	0.0011 (7)	−0.0021 (6)	0.0004 (6)

### Geometric parameters (Å, º)

**Table:**

S1—O2	1.4294 (15)	C5—C6	1.533 (2)
S1—O1	1.4360 (14)	C6—H6A	0.9700
S1—N2	1.6044 (15)	C6—H6B	0.9700
S1—C12	1.7677 (15)	C7—H7A	0.9600
N1—C1	1.356 (2)	C7—H7B	0.9600
N1—C9	1.410 (2)	C7—H7C	0.9600
N1—H1N1	0.88 (2)	C8—H8A	0.9600
N2—H1N2	0.89 (2)	C8—H8B	0.9600
N2—H2N2	0.88 (2)	C8—H8C	0.9600
O3—C3	1.246 (2)	C9—C10	1.389 (2)
C1—C2	1.358 (2)	C9—C14	1.396 (2)
C1—C6	1.505 (2)	C10—C11	1.384 (2)
C2—C3	1.423 (2)	C10—H10A	0.9300
C2—H2A	0.9300	C11—C12	1.383 (2)
C3—C4	1.505 (2)	C11—H11A	0.9300
C4—C5	1.532 (2)	C12—C13	1.385 (2)
C4—H4A	0.9700	C13—C14	1.383 (2)
C4—H4B	0.9700	C13—H13A	0.9300
C5—C8	1.528 (3)	C14—H14A	0.9300
C5—C7	1.532 (2)		
			
O2—S1—O1	118.92 (9)	C5—C6—H6A	108.6
O2—S1—N2	108.30 (10)	C1—C6—H6B	108.6
O1—S1—N2	106.15 (9)	C5—C6—H6B	108.6
O2—S1—C12	106.95 (8)	H6A—C6—H6B	107.6
O1—S1—C12	107.06 (8)	C5—C7—H7A	109.5
N2—S1—C12	109.21 (8)	C5—C7—H7B	109.5
C1—N1—C9	125.62 (14)	H7A—C7—H7B	109.5
C1—N1—H1N1	118.7 (14)	C5—C7—H7C	109.5
C9—N1—H1N1	115.6 (14)	H7A—C7—H7C	109.5
S1—N2—H1N2	106.5 (15)	H7B—C7—H7C	109.5
S1—N2—H2N2	111.5 (16)	C5—C8—H8A	109.5
H1N2—N2—H2N2	121 (2)	C5—C8—H8B	109.5
N1—C1—C2	123.34 (15)	H8A—C8—H8B	109.5
N1—C1—C6	114.23 (14)	C5—C8—H8C	109.5
C2—C1—C6	122.41 (15)	H8A—C8—H8C	109.5
C1—C2—C3	121.35 (15)	H8B—C8—H8C	109.5
C1—C2—H2A	119.3	C10—C9—C14	120.15 (14)
C3—C2—H2A	119.3	C10—C9—N1	120.67 (14)
O3—C3—C2	121.40 (16)	C14—C9—N1	119.09 (15)
O3—C3—C4	121.27 (16)	C11—C10—C9	119.55 (15)
C2—C3—C4	117.33 (15)	C11—C10—H10A	120.2
C3—C4—C5	111.63 (14)	C9—C10—H10A	120.2
C3—C4—H4A	109.3	C12—C11—C10	120.03 (15)
C5—C4—H4A	109.3	C12—C11—H11A	120.0
C3—C4—H4B	109.3	C10—C11—H11A	120.0
C5—C4—H4B	109.3	C11—C12—C13	120.75 (14)
H4A—C4—H4B	108.0	C11—C12—S1	119.01 (12)
C8—C5—C7	109.08 (17)	C13—C12—S1	120.24 (12)
C8—C5—C4	109.87 (17)	C14—C13—C12	119.51 (14)
C7—C5—C4	109.60 (16)	C14—C13—H13A	120.2
C8—C5—C6	110.33 (15)	C12—C13—H13A	120.2
C7—C5—C6	108.87 (15)	C13—C14—C9	119.93 (15)
C4—C5—C6	109.06 (14)	C13—C14—H14A	120.0
C1—C6—C5	114.73 (14)	C9—C14—H14A	120.0
C1—C6—H6A	108.6		
			
C9—N1—C1—C2	14.4 (3)	C1—N1—C9—C14	−139.55 (17)
C9—N1—C1—C6	−167.16 (15)	C14—C9—C10—C11	2.8 (2)
N1—C1—C2—C3	−178.77 (15)	N1—C9—C10—C11	179.57 (14)
C6—C1—C2—C3	2.9 (3)	C9—C10—C11—C12	−0.4 (2)
C1—C2—C3—O3	−166.50 (17)	C10—C11—C12—C13	−1.7 (2)
C1—C2—C3—C4	12.7 (3)	C10—C11—C12—S1	177.15 (12)
O3—C3—C4—C5	136.43 (17)	O2—S1—C12—C11	5.87 (16)
C2—C3—C4—C5	−42.8 (2)	O1—S1—C12—C11	−122.63 (13)
C3—C4—C5—C8	−65.7 (2)	N2—S1—C12—C11	122.86 (14)
C3—C4—C5—C7	174.46 (16)	O2—S1—C12—C13	−175.28 (13)
C3—C4—C5—C6	55.35 (19)	O1—S1—C12—C13	56.22 (15)
N1—C1—C6—C5	−165.84 (15)	N2—S1—C12—C13	−58.29 (15)
C2—C1—C6—C5	12.6 (2)	C11—C12—C13—C14	1.4 (2)
C8—C5—C6—C1	79.9 (2)	S1—C12—C13—C14	−177.40 (12)
C7—C5—C6—C1	−160.42 (16)	C12—C13—C14—C9	1.0 (2)
C4—C5—C6—C1	−40.9 (2)	C10—C9—C14—C13	−3.1 (2)
C1—N1—C9—C10	43.7 (2)	N1—C9—C14—C13	−179.88 (14)

### Hydrogen-bond geometry (Å, º)

**Table:**

*D*—H···*A*	*D*—H	H···*A*	*D*···*A*	*D*—H···*A*
N1—H1*N*1···O1^i^	0.88 (2)	2.10 (2)	2.958 (2)	166 (2)
N2—H1*N*2···O3^ii^	0.89 (2)	2.02 (2)	2.900 (2)	169 (2)
N2—H2*N*2···O3^iii^	0.88 (2)	2.13 (2)	2.969 (2)	159 (2)
C6—H6*A*···O2^iv^	0.97	2.44	3.229 (2)	139

Symmetry codes: (i) −*x*+1/2, *y*−1/2, *z*; (ii) −*x*+1/2, −*y*, *z*+1/2; (iii) *x*−1/2, *y*, −*z*+1/2; (iv) −*x*, *y*−1/2, −*z*+1/2.
